# Increasing Older Adult Involvement in Geriatric Assessment: A Mixed-Methods Process Evaluation

**DOI:** 10.1177/0898264321993321

**Published:** 2021-02-24

**Authors:** Wanda Rietkerk, Jannet de Jonge-de Haan, Joris P. J. Slaets, Sytse U. Zuidema, Debby L. Gerritsen

**Affiliations:** 1 Department of General Practice and Elderly Care Medicine, 3647 University Medical Center Groningen, University of Groningen, Groningen, the Netherlands; 2 Amsterdam University of Applied Science, the Netherlands; 3 Faculty of Medical Sciences, 3647 University Medical Centre Groningen, University of Groningen, Groningen, the Netherlands; 4 443696 Leyden Academy on Vitality and Ageing, Leiden, the Netherlands; 5 Radboud University Medical Center, Radboud Institute for Health Sciences, Nijmegen, the Netherlands

**Keywords:** implementation, motivational interviewing, goal setting, process assessment, preventive health services

## Abstract

**Objectives:** Goal setting and motivational interviewing (MI) may increase well-being by promoting healthy behavior. Since we failed to show improved well-being in a proactive assessment service for community-dwelling older adults applying these techniques, we studied whether implementation processes could explain this. **Methods:** Goals set during the comprehensive geriatric assessment were evaluated on their potential for behavior change. MI and goal setting adherence wasassessed by reviewing audiotaped interactions and interviewing care professionals. **Results:** Among the 280 goals set with 230 frail older adults (mean age 77 ± 6.9 years, 59% women), more than 90% had a low potential for behavior change. Quality thresholds for MI were reached in only one of the 11 interactions. Application was hindered by the context and the limited proficiency of care professionals. **Discussion:** Implementation was suboptimal for goal setting and MI. This decreased the potential for improved well-being in the participating older adults.

## Introduction

It is recommended that clinicians should strive to provide integrated care for older adults (World Health Organization, 2015b). Such care can be provided within proactive comprehensive geriatric assessment services that combine a population screening strategy with an interdisciplinary multi-domain approach (Pilotto et al., 2016). Next to the need for integrated care, older adults and care professionals, each advocate the need for a more person-centered approach to care that increases well-being. This can be achieved by enhancing the involvement of older adults using behavior change and goal identification (Hopman et al., 2016). Motivational interviewing (MI) and goal setting are established methods for delivering these aims.

MI is a well-researched person-centered care communication strategy used by professionals to promote healthy behavior and achieve health benefits (Grandes et al., 2008). It involves a care professional exploring and resolving individual ambivalence to behavior change (Miller & Rollnick, 2014) rather than merely giving a diagnosis and advice. Four overlapping processes are involved: *engaging* (establishing a trusting relationship), *focusing* (determining the target for change), *evoking* (eliciting change talk, i.e., motivational statements about change), and *planning* (increasing commitment to change and formulating an individualized plan of action). The care professional should use adherent strategies for MI (i.e., affirmation, seeking collaboration, and emphasizing autonomy) and avoid non-adherent strategies (i.e., confronting and persuading).

Although MI was developed within psychiatry, it has since then been applied in various care settings, including primary care (Morton et al., 2015). The technique has been shown to be effective in increasing healthy behavior, treatment compliance, motivation, and emotional well-being (Lundahl et al., 2010), including the older adult population (Morton et al., 2015). A key element of MI is to identify goals during the focusing process. Indeed, the identification and setting of goals is considered a key aspect to delivering true person-centered care (World Health Organization, 2015a). Goal setting is also commonly used to increase patient involvement in decision-making and to increase their overall motivation (Levack et al., 2006; Schulman-Green et al., 2006). It has proven feasibility for use with older adults (Robben et al., 2015) and is effective as a behavior change technique (Herdman et al., 2019). The addition of MI and goal setting techniques to existing care could, therefore, help to enhance person-centeredness.

Previously, we incorporated MI and goal setting in a proactive outpatient assessment service for frail community-dwelling older adults, called Sage-atAge+ (in Dutch, *Wijs Grijs 2.0*). Although we had sought to increase well-being through increased patient involvement and behavior change, the inclusion of these techniques had no additional effects on the physical, psychological, or social well-being of older adults (Rietkerk Gerritsen, et al., 2019). However, before concluding that these strategies were ineffective, it is necessary to determine the extent to which they were actually applied. This is because it can be difficult to implement a multicomponent trial into daily practice, because the extent of performance cannot always be known, and because performance of different components may vary (Craig et al., 2008). Studying the extent of performance is crucial to preserve both the internal and external validity, and it can provide invaluable insights into the reasons for an intervention’s success or failure (Leontjevas et al., 2012). This ensures that results are interpreted accurately to facilitate the successful translation of evidence-based interventions into practice (Mackenzie et al., 2010).

The implementation of separate study components should be evaluated alongside the effect evaluation (Mackenzie et al., 2010), thereby allowing conclusions to be made about the efficacy of components and to understand the results of a multicomponent intervention. Understanding the reasons for inadequate implementation rates is important for two main reasons. On the one hand, the components can seem promising, but implementation (strategies) or components may need to be further adapted to the local context to improve implementation (Smit et al., 2018). On the other hand, it may be that further implementation of the intervention and its components add no benefit. Measuring the quality and the extent of MI performance, known as treatment fidelity, is already recognized to be a key factor when appraising trials of MI (Miller & Rollnick, 2014). For goal setting, it is important to determine the extent to which goals have the characteristics needed to promote behavior change (Michie et al., 2011).

Given that MI and goal setting during outpatient assessments could be beneficial for community-dwelling older adults’ well-being through health promotion and person-centeredness, we aimed to study the extent to which these strategies were adopted within Sage-atAge+ and to identify the factors that influenced their use. Since we found no effect on well-being in the Sage-atAge+ program, we hypothesized suboptimal implementation of at least one program component. In order to be able to improve future care programs, we searched for factors explaining this (hypothesized) suboptimal implementation.

## Method

### Design

**Figure 1.**
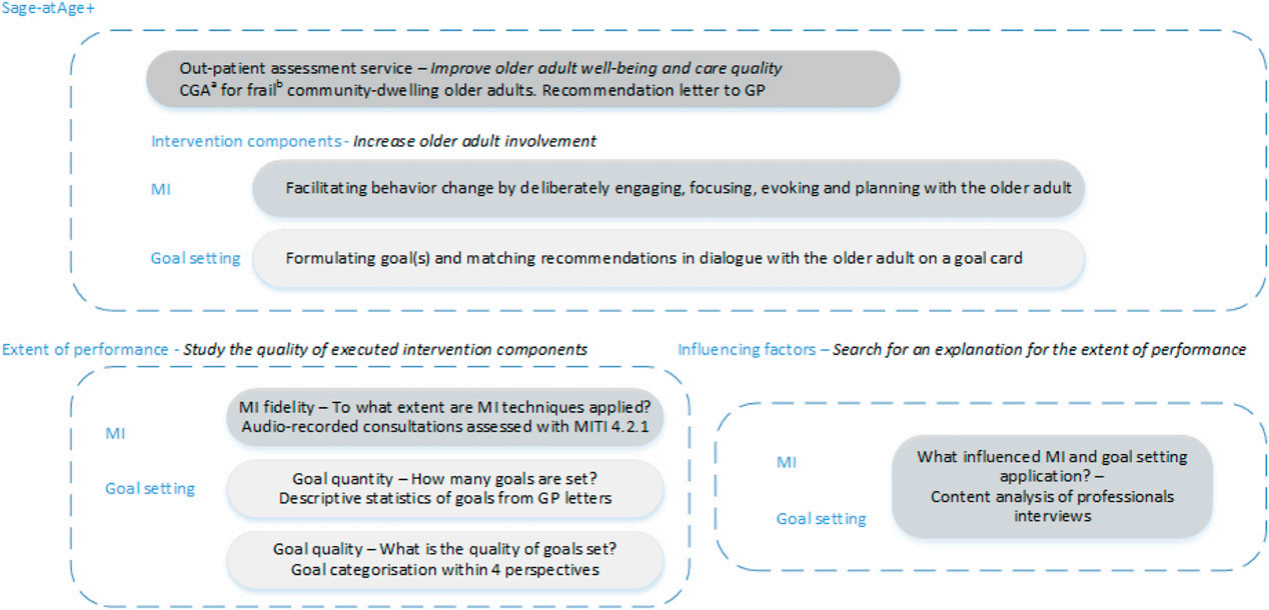
Study design, intervention components, process evaluation questions, and data sources. Notes: *Italic*: Component’s aim. ^a^ comprising an assessment by a nurse or (for the most frail older adults) by an elderly care physician, oral screening by dental care worker, medication evaluation by a pharmacist, and, if applicable, consult from an allied care professional; ^b^ GFI ≥4 (possible range 0–15) (Peters et al., 2012) and/or care profile based on frailty and case complexity ≥2 (possible range 1–5) (Eissens van der Laan et al., 2014). Abbreviations: CGA = comprehensive geriatric assessment; GP = general practitioner; MI = motivational interviewing; MITI = MI Treatment Integrity (code)

We performed a mixed-methods process evaluation alongside our study into the effects of the Sage-atAge+ outpatient assessment service for community-dwelling older adults. This service was designed to increase the general well-being of participants by enhancing their involvement in resolving their unmet needs. We studied the implementation rate of MI and goal setting and their implementation quality (Leontjevas et al., 2012). We also searched for explanations for the extent of performance by analyzing the judgments and experiences of care professionals who had used MI and goal setting. The process analysis focused on the perspectives of care professionals because the perspectives of older adults were thoroughly considered in another study (Rietkerk, Smit, et al., 2019). Figure 1 shows the relation between the intervention components, the questions used in the process evaluation, and the data sources.

### Ethical Considerations

The study was conducted in accordance with the Declaration of Helsinki and the Code of Conduct for Health Research (2004). The Ethics Committee of the University Medical Center Groningen confirmed that the study did not require ethical approval based on the Dutch law for medical trials (M12.120835). Because of the pragmatic nature of the intervention, older adults could also attain the assessment without giving informed consent for scientific data usage. Data in this study were only used from older adults who gave their written informed consent. All care professionals consented to the publication of the final manuscript.

### Sage-atAge+ Service

The Sage-atAge+ service comprised two steps: (1) proactive screening of community-dwelling older adults for frailty and case complexity; and (2) assessment of needs and goals of older adults identified as frail. We then generated recommendations for the frail older adult and their general practitioner (GP). Older adult involvement in the intervention was promoted by using MI and goal setting techniques.

Patients were selected for assessment by sending a postal questionnaire and informed consent form to 1495 adults aged ≥65 years from seven primary care practices in the rural Northern part of the Netherlands. In total, 49% of the older adults (*n* = 725) returned the questionnaires. The questionnaires contained the Groningen Frailty Indicator (GFI) (Peters et al., 2012) and self-assessment version of the INTERMED for the elderly (Peters et al., 2013), respectively. With these, care profiles were attained as follows: (1) feeling vital, (2) psychosocial coping difficulties, (3) physical and mobility needs, and (4) difficulties in multiple domains (Eissens van der Laan et al., 2014). These profiles were constructed in previous research by factor mixture model analysis and were used to adapt the service to patient needs. Older adults with a substantial frailty level (GFI ≥ 4) and/or a high care profile (≥2) (44% (*n* = 322)) were invited for a CGA between September 1, 2014, and April 1, 2016. Overall, their mean age was 77 ± 6.9 years (range 65–94), 59% were women, 60% were married, 33% had a low educational level ((uncompleted) primary school or low-level vocational training), and 97% were of Dutch ethnicity. Mean frailty as measured with the GFI was 4.5 (±2.2). Details of the recruitment and selection procedure, as well as the participant characteristics, are published elsewhere (Rietkerk, Gerritsen, et al., 2019).

### Comprehensive Geriatric Assessments

Comprehensive geriatric assessments were provided by a nurse or an elderly care physician (Koopmans et al., 2017), with the latter only performing assessments for the most complex and frail cases. The focus of these assessments was well-being, including social and functional participation, physical and psychological needs, and the living situation. A pharmacist performed a risk assessment of drug-related problems based on the triage score system (Van Roon et al., 2007) and the structured history-taking of medication use tool (Drenth-Van Maanen et al., 2011) and a dental care worker took an oral history and assessed the oral cavity. If considered necessary, optional diagnostic consultations could be added from a dietitian, physiotherapist, psychologist, or occupational therapist.

Written summaries of the assessments, consisting of one or more points of concern, corresponding life and health-related goals, and recommendations, were formulated and written on a *goal card* in collaboration with the older adult. MI was used to stimulate the older adult’s ability to reach the goals by deliberately improving their motivation through engaging, focusing, evoking, and planning. The content of the *goal card* was recorded in the older adult’s file and incorporated in the letter to his or her GP.

Between October 2014 and January 2015, all care professionals involved in the Sage-atAge+ study were invited for three 4-hour training sessions about MI. During these, didactic instruction was combined with role playing to allow practice with eliciting change talk, seeking collaboration, and goal setting. Participants received instructor and peer feedback on their performance. To improve compliance and sustained adoption of the newly acquired techniques, two booster sessions were held with care professionals that reinforced the training and provided an opportunity to discuss practical experiences.

### MI Fidelity of Audio-Recorded Interactions

The MI fidelity was rated with the Motivational Interviewing Treatment Integrity (MITI) coding system (Moyers et al., 2014). The MITI is a reliable behavioral coding system which assesses the extent to which MI techniques are used during interactions by coding the remarks of care professionals. We audiotaped and transcribed 11 consecutive assessments by care professionals during December 2016 (four times by a nurse, three times by a pharmacist, and once each by an elderly care physician, a physiotherapist, a psychologist, and a dietitian). The median assessment length was 32 (range 16–65) minutes. Following the MITI, a 20-minute segment of each session was coded independently by two students studying for a master’s qualification in a health field. Whenever the audio-recorded assessments exceeded 20 minutes, a segment of that length was selected that focused on a target change behavior. Both coders had received 20 hours of training in coding with the MITI by an expert from the MI network of trainers (JJ) who also provided supervision while they performed the coding.

First, we scored four global MI qualities (i.e., partnership, empathy, cultivating change talk, and softening sustain talk) on a Likert scale that captured the coder’s overall judgment of the global qualities of an interaction. Summary scores were calculated with these and checked against expert-derived *fair* thresholds, which are considered the minimum extent of MI application needed to obtain the desired effects (Moyers et al., 2016):

1. The *relational score* is the average of the *partnership* and *empathy* global scales. Higher scores indicate clinicians trying to foster a collaborative approach and genuinely seeking to understand a patient’s perspective. The possible range is 1–5, with the *fair* threshold set at 3.5.
2. The *technical score* is the average of the *softening sustain talk* and *cultivating change talk global scores*. Higher scores indicate clinicians actively eliciting the patient’s arguments in favor of positive change (i.e., change talk) and decreasing the patient’s arguments for no change (i.e., sustain talk). The possible range is 1–5, with the *fair* threshold set at 4.
3. The *reflection to question ratio* is calculated, with the fair threshold set to a ratio of one reflection to one question. Higher scores indicate that the clinician centers on engagement and evocation. The *fair* threshold is set at one or more reflections to one question.
4. We calculated the *percentage of complex reflections* compared to the sum of complex and simple reflections. The fair threshold was set as >40%.

Each relevant utterance of a care professional was counted as an adherent (i.e., affirming, seeking collaboration, and emphasizing autonomy) or a non-adherent (i.e., confronting and persuading) behavior category of MI.

### Goal Setting: Quantity and Quality Based on Medical Records

The recommendation letter to the GP was extracted from all participants’ medical records, and any goal(s) were recorded, if applicable. *Goal quantity* was described by the median number of goals per participant with the interquartile range (IQR) and total range. *Goal quality* was classified into four categories: desire language, goal level, magnitude specification, and time frame specification.

#### Desire language

We coded this language element because it is known to be associated with the potential for behavior change (Tonigan et al., 2018). Every goal using the words “want,” “desire,” “like,” or a synonym of those words was coded as desire language (e.g., “would like to improve walking”) (Miller et al., 2008).

#### Goal level

We classified goals at the behavior or the outcome level based on an adaptation of a taxonomy for behavior change techniques (Michie et al., 2011). Goals at the *behavior level* were those targeting behavioral solutions (e.g., “playing billiards again; going outside with the mobility scooter”), whereas goals at the *outcome level* were defined in terms of an expected consequence of one or more behaviors, without being a behavior in itself (e.g.*, “*decrease in abdominal discomfort”; “stay independent for as long as possible”). In addition to the taxonomy of Michie et al., to improve our agreement on this discrimination, we coded as the behavior level when a goal could be a task that could be added to a to-do list. Whenever this seemed impossible or if we needed to give more specification on the next step (e.g., “being among people more frequently”), we coded as the outcome level.

#### Specification categories

Specificity was coded because it is considered to increase the potential for behavior change by increasing commitment to that change (Kaminer et al., 2018). These elements were adapted from a taxonomy of important goal elements for people with dementia (Bogardus et al., 1998). *Magnitude* was coded into three categories: *magnitude or volume specified* if the goal was objectively measurable (e.g., “having a daily walk”), *mentioned but not quantified* if the goal was specified without the amount (e.g., “stabilize weight loss”), and *not mentioned* if the evaluation criterion for a goal was unknown (e.g., “decrease stress”). *Time frame* was also coded into three categories, as follows: *specified* if the time to attain the goal could be measured on a calendar, *mentioned but not specified* if a vague time period was used (e.g., “soon or as long as possible”), and *not mentioned* if no time period was given.

### Interrater Agreement

The interrater agreement for MITI coding was assessed on five recordings between the two coders with a two-way mixed effects model, absolute agreement, and average measures intra-class correlation coefficient. The mean interrater agreement for coding between the reviewers was excellent (interclass correlation coefficient 0.81 ± 0.15). All interclass correlation coefficients ranged between good for complex reflections (0.64) and excellent for affirmations (0.95), except for giving information, which was only fair (0.44) (Cicchetti & Sparrow, 1981). When applicable, the average of both raters’ scores was calculated. Finally, we calculated the means, *SD*s, and ranges for the behavior counts and summary scores. The number of interactions reaching the *fair* threshold was counted.

Two researchers (WR and CN) independently applied the taxonomy for goal setting, and their allocation was similar in 94% of categories (range 92%–95%). Cohen’s kappa was 0.48 for the goal level, and the linear Cohen’s kappa values were 0.79 for *magnitude* and 0.83 for *time frame*. Whenever there was disagreement over categorization, consensus was reached after discussion between WR and CN.

### Identification of Influencing Factors

Influencing factors for applying MI and goal setting were identified based on interviews. Semi-structured interviews with all care professionals were held by WR, a physician, and researcher, within 3 months after the Sage-atAge+ program had ended. The topic list comprised questions about the feasibility and acceptability of the program and its intervention components and experience with various elements of the program (e.g., training, MI, goal setting, and goal cards). All interviews were audiotaped, but technical problems resulted in two tapes being unusable. These interviews were transcribed in detail by the interviewer and checked by the interviewee within a week. All other tapes were transcribed verbatim.

We used inductive content analysis to derive findings by focused evaluation of questions phrased by WR and DG (Elo & Kyngäs, 2008). The analysis of transcripts was supported by the software package Atlas.ti 7. WR analyzed all interviews, and discussed themes, and corresponding quotes with JJ and DG regularly. All identified experiences and influencing factors were substantiated with relevant quotes.

## Results

### Sample: Care Professionals and Assessments

Overall, 322 comprehensive geriatric assessments were performed as part of the Sage-atAge+ program. Of these, 29% (*n* = 92) were excluded from analysis due to a lack of informed consent (*n* = 79) or missing medical record data (*n* = 13). Thus, the medical records of 230 participants were available and included in the analysis. All assessments were executed by three specialist geriatric nurses, except for 7% (*n* = 15), which were performed by the elderly care physician. Additional assessments by the pharmacist and the dental care assistant, which were offered to all participants, were undertaken by 94% (*n* = 217) and 29% (*n* = 67), respectively. Consultations with other allied healthcare professionals were attended by 25% (*n* = 57). Finally, a *goal card* was provided during 53% (*n* = 121) of the assessments.

Among the 10 professionals involved in the Sage-atAge+ program, four attended all training sessions, one attended only one session, two attended no sessions (logistical reasons), and three reported that they had already received training in MI. All participants were interviewed, except for the occupational therapist due to logistical reasons. An overview of data about the care professionals, the number of assessments, and their attendance at MI training is presented in Supplementary Table S1.

### Extent of Performance: MI Fidelity

The results of the MITI assessment are shown in Table 1. Adherent behavior was expressed twice on average per interaction (*SD* 1.2, range 0.5–4): specifically, affirmation was expressed once on average per interaction (*SD* 1.0, range 0–2.5), whereas seeking collaboration or emphasizing autonomy was counted a maximum of once. Non-adherent behavior was more common, occurring up to a maximum of 13 times (mean 5.1, *SD* 4.1): confronting was never seen, but persuasion without permission occurred to a maximum of 13 behavior counts in one interaction.

**Table 1.** MITI Coding Results of Audiotaped Interactions (*n* = 11).

	Mean	SD	Range, min–max	Possible Range	Threshold^a^	Threshold Reached, *n* (%)
Behavior counts						
MI adherent behavior, total	2.0	1.2	0.5–4			
Affirm	1.1	1.0	0–2.5	≥0		
Seek collaboration	0.5	0.5	0–1	≥0		
Emphasize autonomy	0.4	0.4	0–1			
MI non-adherent behavior, total	5.1	4.1	0–13.5			
Confront	0.0	0.0	0–0	≥0		
Persuade	5.1	4.1	0–13.5	≥0		
Summary measures						
Relational	3.4	1.0	1.3–4.5	1–5	≥3.5	8 (73)
Technical	3.2	0.6	1.5–4	1–5	≥3	10 (91)
Reflection to question ratio	0.8	0.6	0.1–2	>0	≥1	4 (36)
% Complex reflections	20	2.0	0–50	0–100	≥40	1 (9)

Abbreviations: MI = motivational interviewing; MITI = MI Treatment Integrity code 4.2.1; SD = standard deviation.

^a^The fair threshold was used (Theresa B Moyers et al., 2014).

Concerning the summary measures, one interaction met none of the four thresholds and only one interaction met all four thresholds. The threshold on the *relational scale* was met in eight interactions, whereas that on the *technical global scale* was met in all but one. The *reflection to question ratio* was above the threshold in four of 11 interactions. Only in one interaction was the threshold for the *complex reflection ratio* reached.

### Extent of Performance: Goal Setting

In 206 of the 230 assessments (90%), 280 goals were formulated by the geriatric nurses or the elderly care physician and presented in a recommendation letter to a GP. The median goal count per adult was 1 (IQR 1–2; range 1–4). Goals mostly aimed to preserve the status quo (51%, *n* = 144), with the most common goals being to preserve independence or self-sufficiency for as long as possible (*n* = 30) and to preserve mobility for as long as possible (*n* = 28). The allocation of all goals among the four characteristics is shown in Table 2. Desire talk was used in 21% of the goals, the behavior level was specified in 9%, and the *magnitude* was specified in 4%. *Time frame* was specified in only one goal.

**Table 2.** Goal Quality From Four Different Perspectives (*n* = 280).

Quality perspective	Category	*n* (%)
Desire language		
	Used	60 (21)
	Not used	220 (79)
Goal level		
	Behavior	26 (9)
	Outcome	254 (91)
Specificity–magnitude		
	Specified	10 (4)
	Mentioned but not quantified	28 (10)
	Not mentioned	242 (86)
Specificity–timeframe		
	Specified	1 (0)
	Mentioned but not quantified	142 (51)
	Not mentioned	137 (49)

Abbreviations: NA = not applicable.

### Influences on Applying MI and Goal Setting

**Figure 2.**
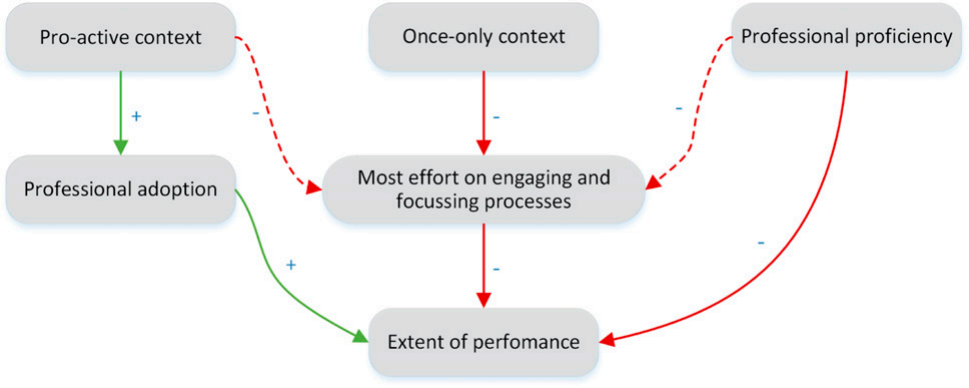
Factors influencing the extent of performance of MI and goal setting within a proactive assessment program. Legend: green lines = positive influence; red lines = negative influence; solid line = strongly substantiated within our data; dashed line = moderately substantiated within our data. Abbreviations: MI = motivational interviewing.

The interviews revealed that the extents to which MI and goal setting were applied were influenced by the context and the care professional’s proficiency. Moreover, not all MI processes were sufficiently applied and are described in Figure 2 and in detail in the following text.

#### Context: Sage-atAge+ was proactive and once only

Care professionals expressed the need to increase participant motivation because of the proactive approach. However, because the older adults did not volunteer for the service, they experienced low ownership, did not expect benefit, felt no urgency, and started with a passive attitude. This resulted in care professionals needing to focus on overcoming significant motivational barriers.

“Since you start from scratch in this visit, both for yourself and the one in front of you, one hour might not be enough to create internal motivation. So, during the conversation something will come up, but they also need to be motivated. “..” Only when it is something urgent, something they would have wanted to do for a long time, it will succeed.” (Elderly care physician)

Thus, there was high motivation among the care professionals to adopt MI techniques and goal setting. They believed these techniques could be of real benefit in engaging older adults and helping them to formulate and reach their goals.

“[Sage-atAge+] is a supplement for frail older adults since they take their frailty for granted most of the time, or they don’t see it. By having a conversation with them, many things will come up. Things that they do not discuss with their [GP] or that just had slipped in.” (Dental care worker)

The proactive approach not only influenced the motivation of care professionals but also made engagement a delicate process. This was because the professionals often felt less authority and experienced friction during the engaging process.

“At a certain moment you feel when people are having resistance, that you are meddling some one’s autonomy. I don’t think that is ok. “..” As a screener, without having a relationship with the patient, I should not cross that line.” (Nurse B)

The once-only context negatively influenced whether MI and goal setting were applied, often leaving the professionals feeling reluctant to engage in depth, specifically regarding psychological topics.

“At some moments I was wary to get an intimate discourse by asking a certain question. And then I would never see them again. Yes, that is definitely a disadvantage.” (Nurse A)

The once-only context also made it difficult to reflect on earlier attempts and engagement with health behavior change, which hampered the evoking process.

“That is also a disadvantage of seeing them only once. Otherwise you could ask them whether they succeeded or what challenges they experienced, since that is something we can work on together.” (Nurse C)

#### Care professional’s proficiency

Suboptimal proficiency in MI negatively influenced effective delivery. For example, the evoking process was hampered because some care professionals feared achieving an opposite result when triggering sustain talk.

“It could be a sensitive issue for people, especially if you give them the feeling they are not performing well. “..” And since I did not know how to deal with that, I avoided the topic, to prevent saying the wrong things and making them grab their cigarettes. I did not feel familiar with that, so I preferred to avoid these topics.” (Nurse C)

Most care professionals expressed a need for ongoing training or booster sessions to improve their knowledge and to exchange their experiences (e.g., dealing with barriers).

“And we also discussed the way we wrote stuff down [on the goal cards]. “..” You can consult each other … that would have been good, and I think it has been done too little, since that is something you can learn from.” (Nurse A)

#### Most effort was spent on the engaging and focusing processes

Professionals felt they lacked the time to execute all MI processes because of the need to overcome the barrier of low participant motivation during assessments.

“You need to become acquainted with people, you need to win their trust, you are talking about what they have raised in the questionnaire, and you hope that they will find some motivation to start working on things that might have been considered obvious but could become a problem. That is a lot to do within a one-hour visit.” (Nurse B)

When mentioning a follow-up context, however, less time was spent on the engaging and focusing processes of MI.

“And you notice, when people come for a follow-up visit, they will get back to the first conversation, and then it is easier to continue with what has been discussed before. It is easier to step in, so to speak. Because you have already won their trust.” (Nurse B)

The limited proficiency with MI of care professionals, especially with the evoking process, meant they felt more comfortable with the engaging and focusing processes.

“For example, a man who is smoking and does not consider this an issue. At these moments I think ‘never mind, who am I to say something about this?’” (Nurse C)

Overall, the extents of performing MI and goal setting were positively influenced by the high motivation and attempted adoption by professionals. However, although the Sage-atAge+ context contributed to this motivation, it also hampered the extent of performance mostly because a lot of effort needed to be spent on the engaging and focusing processes. We found that the proficiency of participating care professionals was insufficient to overcome these barriers.

## Discussion

Implementation research is essential for the successful translation of evidence-based interventions into practice. Using a mixed-methods strategy, we studied the extent to which MI and goal setting were performed within the Sage-atAge+ proactive assessment program for community-dwelling older adults (65+) to identify why we failed to show the hypothesized effect on well-being (Rietkerk, Gerritsen, et al., 2019). In the current study, we showed that goal setting was prevalent, but that MI fidelity and the quality of goals were suboptimal, despite high motivation by care professionals. Overall, the proactive and once-only context of the service, as well as the limited proficiency of the care professionals, were the main factors hindering successful implementation.

MI fidelity was low in our research, with only one in 11 interactions reaching all thresholds for which effect on behavior change was expected. Adherent behavior was also infrequent during the motivational interviews, despite its known effectiveness for improving healthy behaviors (Tonigan et al., 2018). When compared to GPs without training in MI (McKenzie et al., 2021), the consultations in our study had much better scores on the *relational* and *technical summary scores*, implying these care professionals performed better at engaging with their patients and in delivering patient-centered or patient-friendly consulting. However, the professionals did report limited proficiency, especially when evoking change talk, which is consistent with reports that it is the most difficult MI skill to acquire (Resnicow & McMaster, 2012). Limited proficiency is often seen in trials of MI (Forsberg et al., 2017) and can be attributed both to limited training and the need to update and consolidate skills over time through booster sessions (Miller & Rollnick, 2014).

Goal setting was highly implemented, with 90% of all letters to GPs containing at least one goal for which desire talk was prevalent. The mapping of these individual needs and wishes within goals is central to person-centered care (World Health Organization, 2015a) and was reflected in the positive attitudes of participants toward the service (Rietkerk, Smit, et al., 2019). However, only a minority of the formulated goals contained aspects that were expected to increase the potential for behavior change. The fact that professionals spent most effort and time in engaging and focusing participants meant that less time was available to specify goals further or to elaborate on goal planning.

Most goals that we identified were aimed at maintaining the status quo and not at delivering tangible improvements, which is in line with other recent research on proactive goal setting with community-dwelling older adults (Javadi et al., 2018). The fact that we detected preventive or long-term needs rather than urgent needs likely results from the proactive approach of Sage-atAge+. However, to achieve the actual benefits of preventive behavior change, much more effort is needed for goal planning compared to the requirement of goals that seek to achieve short-term benefits, especially for older people (Hall & Fong, 2007).

The proactive and once-only context of Sage-atAge+ was probably the main reason for care professionals spending most of their interaction time on the engaging and focusing processes of MI, and having very little time for evoking and planning. This represented the care professionals adapting to the needs of older adults, who often first required to set goals, before discussing their ambivalence for behavior change and starting action planning. However, by failing to complete all processes, the necessary tools to achieve meaningful change were not delivered. If a professional is to complete an assessment after only achieving engagement and focus, follow-up will be needed to specify goals through evoking and planning. This could explain the limited effect of any outpatient assessment service that lacks direct influence over the implementation of recommendations (Chen & Steinman, 2016). The modifying effect of this influence has also been reported in earlier reviews on the effects of outpatient assessment services (Pilotto et al., 2016). To date, we are unaware of any studies describing the role of failure to execute the goal planning process on the limited impact of those services.

## Methodological Considerations

Some remarks can be made about the methods and validity of the current study. The mixed-methods strategy allowed us to explain the results of implementation, with the quantitative results complementing the qualitative results. Together, they formed the basis of our conclusion that suboptimal MI and goal setting explained the failure to achieve the hypothesized outcomes of the Sage-atAge+ program.

We complied with the criteria proposed by Jelsma et al. when coding and reporting the MI fidelity (Jelsma et al., 2015) but not with the minimum recommended collection of 20 interactions. Although this could have hampered the validity of our assessment of MI fidelity, it should be noted that we substantiated our fidelity findings by including the experiences of care professionals and by assessing the quality of goal setting. Consequently, we expect our conclusion about suboptimal MI to be valid.

We are not aware of a feasible goal taxonomy for coding comprehensive goal quality, consistent with an existing report that currently available goal setting evaluation tools are inadequate (Vermunt et al., 2017). Therefore, we combined several existing goal characteristic classifications from the literature. By doing so, we created a taxonomy that was feasible and had high agreement and reasonable kappa scores. The discussions between raters led to full agreement. With this taxonomy, we could provide insights into the characteristics and quality of goals, the proficiency of care professionals in goal setting, and the potential of goals to result in desired effects. However, external validation is needed before we can advocate further usage of this taxonomy.

It should also be noted that the care professionals who participated in this research did not receive specific training on the goal characteristics we reviewed with the taxonomy, which may have contributed to the low quality we found. In addition, this gives a useful insight into the reality of adding goal setting to daily practice without specific training.

## Recommendations for future research and practice

Our current study results help to explain not only the extent of performance (i.e., how the older adult involvement was enhanced within the outpatient proactive assessment service) but also the factors that influenced that extent of performance. This may lead to service enhancements and adaptations, including the addition of behavior change techniques (Michie et al., 2011). For example, by allowing for follow-up to deliver goal planning services, we may improve participant engagement and increase effect on well-being. Equally, the implementation of MI could be enhanced to increase fidelity: this may involve extending the initial training, offering booster sessions, or better adapting to the needs of professionals by focusing on the process of evoking. Fidelity could even be monitored during service delivery to control for whether MI thresholds are reached.

In terms of future research goals, the impact of proactive approaches on participant engagement requires further study. It could be tested whether this is a key barrier in other integrated proactive programs for older adults. Such research into implementation may improve our understanding of the additional value of MI strategies within person-centered care for older adults. Designs can be improved to overcome the barriers to motivation and goal planning, such as implementing case management instead of once-only assessment strategies. However, if we are to deliver true person-centered care, we must avoid striving blindly for behavior change if it is at the expense of recognizing the goals of the older adult, who may not want behavior change.

## Conclusions

In this mixed-methods implementation study, we aimed to identify the reasons for failure to achieve improved well-being in a previous study (Rietkerk, Gerritsen, et al., 2019). We found that MI fidelity and goal quality were suboptimal despite a high prevalence of goal setting. Several issues contributed to these problems. It is true that care professionals lacked some proficiency with MI, especially with the evoking process, resulting in less time being spent on evoking and planning, and decreasing the opportunity to resolve ambivalence to behavior change among the older adults. However, our findings indicate that this was not the full extent of the problem. Perhaps of even greater importance was the proactive and once-only context of the Sage-atAge+ service. To improve MI and goal setting implementation in the future, we should not only seek to focus on adding booster training sessions but also on adopting a case management approach that allows for adequate patient follow-up over multiple sessions. Lessons learned from implementation studies that are conducted alongside effect evaluations can help both to improve care and to develop effective and efficient care programs.

## Supplemental Material

sj-pdf-1-jah-10.1177_0898264321993321 – Supplemental Material for Increasing Older Adult Involvement in Geriatric Assessment: A Mixed-Methods Process Evaluation Click here for additional data file. Supplemental Material, sj-pdf-1-jah-10.1177_0898264321993321 for Increasing Older Adult Involvement in Geriatric Assessment: A Mixed-Methods Process Evaluation by Wanda Rietkerk, Jannet de Jonge-de Haan, Joris P. J. Slaets, Sytse U. Zuidema and Debby L. Gerritsen in Journal of Aging and Health

## References

[bibr1-0898264321993321] Bogardus S. T. Bradley E. H. Tinetti M. E. (1998). A taxonomy for goal setting in the care of persons with dementia. Journal of General Internal Medicine, 13(10), 675-680. doi:10.1046/j.1525-1497.1998.00203.x 9798814 PMC1500896

[bibr2-0898264321993321] Chen P. Steinman M. A. (2016). Perception of primary care physicians on the impact of comprehensive geriatric assessment: What is the next step? Israel Journal of Health Policy Research, 5(1), 3-6. doi:10.1186/s13584-016-0106-3 27733902 PMC5045624

[bibr3-0898264321993321] Cicchetti D. V Sparrow S. A. (1981). Developing criteria for establishing interrater reliability of specific items: Applications to assessment of adaptive behavior. American Journal of Mental Deficiency, 86(2), 127-137. 7315877

[bibr4-0898264321993321] Craig P. Dieppe P. Macintyre S. Michie S. Nazareth I. Petticrew M. Petticrew M. (2008). Developing and evaluating complex interventions: New guidance. BMJ (Clinical Research Ed.), 337, a1655. doi:10.1136/bmj.a1655 PMC2769032 18824488

[bibr5-0898264321993321] Drenth-Van Maanen A. C. Spee J. Van Marum R. J. Egberts T. C. G. (2011). Structured history taking of medication use reveals iatrogenic harm due to discrepancies in medication histories in hospital and pharmacy records. Journal of the American Geriatrics Society, 59, 1976-1977. doi:10.1111/j.1532-5415.2011.03610_11.x 22091519

[bibr6-0898264321993321] Eissens van der Laan M. R. van Offenbeek M. A. G. Broekhuis H. Slaets J. P. J. (2014). A person-centred segmentation study in elderly care: Towards efficient demand-driven care. Social Science and Medicine, 113, 68-76. doi:10.1016/j.socscimed.2014.05.012 24852657

[bibr7-0898264321993321] Elo S. Kyngäs H. (2008). The qualitative content analysis process. Journal of Advanced Nursing, 62(1), 107-115. doi:10.1111/j.1365-2648.2007.04569.x 18352969

[bibr8-0898264321993321] Forsberg L. Ghaderi A. Diez M. Enö Persson J. Beckman M. Lindqvist H. (2017). The dissemination of motivational interviewing in Swedish county councils: Results of a randomized controlled trial. Plos One, 12(7), e0181715. doi:10.1371/journal.pone.0181715 28750067 PMC5531530

[bibr9-0898264321993321] Grandes G. Sanchez A. Cortada J. M. Balague L. Calderon C. Arrazola A. Millan E. (2008). Is integration of healthy lifestyle promotion into primary care feasible? Discussion and consensus sessions between clinicians and researchers. BMC Health Services Research, 8, 213. doi:10.1186/1472-6963-8-213 18854033 PMC2577098

[bibr10-0898264321993321] Hall P. A. Fong G. T. (2007). Temporal self-regulation theory: A model for individual health behavior. Health Psychology Review, 1(1), 6-52. doi:10.1080/17437190701492437

[bibr11-0898264321993321] Herdman K. A. Vandermorris S. Davidson S. Au A. Troyer A. K. Herdman K. A. Davidson S. (2019). Comparable achievement of client-identified, self-rated goals in intervention and no-intervention groups: Reevaluating the use of goal attainment scaling as an outcome measure. Neuropsychological Rehabilitation, 29(10), 1600-1610. doi:10.1080/09602011.2018.1432490 29430998

[bibr12-0898264321993321] Hopman P. de Bruin S. R. Forjaz M. J. Rodriguez-Blazquez C. Tonnara G. Lemmens L. C. Rijken M. (2016). Effectiveness of comprehensive care programs for patients with multiple chronic conditions or frailty: A systematic literature review. Health Policy, 120(7), 818-832. doi:10.1016/j.healthpol.2016.04.002 27114104

[bibr13-0898264321993321] Javadi D. Lamarche L. Avilla E. Siddiqui R. Gaber J. Bhamani M. Dolovich L. (2018). Feasibility study of goal setting discussions between older adults and volunteers facilitated by an eHealth application: Development of the Health TAPESTRY approach. BMC Pilot and Feasibility Studies, 4(184), 1-13. 10.1186/s40814-018-0377-2 PMC6292127 30564435

[bibr14-0898264321993321] Jelsma J. G. M. Mertens V. Forsberg L. Forsberg L. (2015). How to measure motivational interviewing fidelity in randomized controlled trials: Practical recommendations. Contemporary Clinical Trials, 43, 93-99. doi:10.1016/j.cct.2015.05.001 25962891

[bibr15-0898264321993321] Kaminer Y. Ohannessian C. M. C. McKay J. R. Burke R. H. Flannery K. (2018). Goal commitment predicts treatment outcome for adolescents with alcohol use disorder. Addictive Behaviors, 76, 122-128. doi:10.1016/j.addbeh.2017.07.035 28800496 PMC6490181

[bibr16-0898264321993321] Koopmans R. T. C. M. Pellegrom M. van der Geer E. R. (2017). The Dutch move beyond the concept of nursing home physician specialists. Journal of the American Medical Directors Association, 18(9), 746-749. doi:10.1016/j.jamda.2017.05.013 28668662

[bibr17-0898264321993321] Leontjevas R. Gerritsen D. L. Koopmans R. T. C. M. Smalbrugge M. Vernooij-Dassen M. J. F. J. (2012). Process evaluation to explore internal and external validity of the “Act in Case of Depression” care program in nursing homes. Journal of the American Medical Directors Association, 13(5), 488.e1-8. doi:10.1016/j.jamda.2012.03.006 22521629

[bibr18-0898264321993321] Levack W. M. M. Dean S. G. Siegert R. J. McPherson K. M. (2006). Purposes and mechanisms of goal planning in rehabilitation: The need for a critical distinction. Disability and Rehabilitation, 28(12), 741-749. doi:10.1080/09638280500265961 16754571

[bibr19-0898264321993321] Lundahl B. W. Kunz C. Brownell C. Tollefson D. Burke B. L. (2010). A meta-analysis of motivational interviewing: Twenty-five years of empirical studies. Research on Social Work Practice, 20(2), 137-160. doi:10.1177/1049731509347850

[bibr20-0898264321993321] Mackenzie M. O’Donnell C. Halliday E. Sridharan S. Platt S. (2010). Do health improvement programmes fit with MRC guidance on evaluating complex interventions? British Medical Journal, 340(feb01 1), c185. doi:10.1136/bmj.c185 20123834

[bibr21-0898264321993321] McKenzie K. J. Pierce D. Mercer S. W. Gunn J. M. (2021). Do GPs use motivational interviewing skills in routine consultations with patients living with mental-physical multimorbidity? An observational study of primary care in Scotland. Chronic Illness, 17(1), 29-40. doi:10.1177/1742395318815960 30580557

[bibr22-0898264321993321] Michie S. Ashford S. Sniehotta F. F. Dombrowski S. U. Bishop A. French D. P. (2011). A refined taxonomy of behaviour change techniques to help people change their physical activity and healthy eating behaviours: The CALO-RE taxonomy. Psychology and Health, 26(11), 1479-1498. doi:10.1080/08870446.2010.540664 21678185

[bibr23-0898264321993321] Miller W. R. Moyers T. B. Ernst D. Amrhein P. (2008). Manual for the motivational interviewing skill code (MISC). Version 2.5. Retrieved March 7, 2019, from http://casaa.unm.edu/download/misc.pdf.

[bibr24-0898264321993321] Miller W. R. Rollnick S. (2014). The effectiveness and ineffectiveness of complex behavioral interventions: Impact of treatment fidelity. Contemporary Clinical Trials, 37(2), 234-241. doi:10.1016/j.cct.2014.01.005 24469237

[bibr25-0898264321993321] Morton K. Beauchamp M. Prothero A. Joyce L. Saunders L. Spencer-Bowdage S. Pedlar C. (2015). The effectiveness of motivational interviewing for health behaviour change in primary care settings: A systematic review. Health Psychology Review, 9(2), 205-223. doi:10.1080/17437199.2014.882006 26209209

[bibr26-0898264321993321] Moyers T. B. Rowell L. N. Manuel J. K. Ernst D. B. Houck J. M. (2016). The motivational interviewing treatment integrity code (MITI 4): Rationale, prelimanary reliability and validity. Journal of Substance Abuse Treatment, 65, 1-7. doi:10.1016/j.jsat.2016.01.001 26874558 PMC5539964

[bibr27-0898264321993321] Moyers T. B. Manuel J. K. Ernst D. (2014). De Nederlandse vertaling van de Motivational interviewing treatment integrity Coding Manual 4.2.1 (MITI 4.2.1).

[bibr28-0898264321993321] Peters L. L. Boter H. Buskens E. Slaets J. P. J. (2012). Measurement properties of the groningen frailty indicator in home-dwelling and institutionalized elderly people. Journal of the American Medical Directors Association, 13(6), 546-551. doi:10.1016/j.jamda.2012.04.007 22579590

[bibr29-0898264321993321] Peters L. L. Boter H. Slaets J. P. J. Buskens E. (2013). Development and measurement properties of the self assessment version of the INTERMED for the elderly to assess case complexity. Journal of Psychosomatic Research, 74(6), 518-522. doi:10.1016/j.jpsychores.2013.02.003 23731750

[bibr30-0898264321993321] Pilotto A. Cella A. Pilotto A. Daragjati J. Veronese N. Musacchio C. Panza F. (2016). Three decades of comprehensive geriatric assessment: Evidence coming from different healthcare settings and specific clinical conditions. Journal of the American Medical Directors Association, 18(2), 192.e1-192.e11. doi:10.1016/j.jamda.2016.11.004 28049616

[bibr31-0898264321993321] Resnicow K. McMaster F. (2012). Motivational interviewing: Moving from why to how with autonomy support. International Journal of Behavioral Nutrition and Physical Activity, 9(1), 19. doi:10.1186/1479-5868-9-19 PMC3330017 22385702

[bibr32-0898264321993321] Rietkerk W. Gerritsen D. L. D. Kollen B. J. Hofman C. S. Wynia K. Slaets J. P. J. Zuidema S. U. (2019). Effects of increasing the involvement of community-dwelling frail older adults in a proactive assessment service: A pragmatic trial. Clinical Interventions in Aging, 14, 1985-1995. doi:10.2147/CIA.S206100 31814713 PMC6858288

[bibr33-0898264321993321] Rietkerk W. Smit M. F. Wynia K. Slaets J. P. J. Zuidema S. U. Gerritsen D. L. (2019). Explaining experiences of community- dwelling older adults with a pro-active comprehensive geriatric assessment program – a thorough evaluation by interviews. BMC Geriatrics, 19(12), 1-13. 30642257 10.1186/s12877-018-1025-7 PMC6332689

[bibr34-0898264321993321] Robben S. H. M. Heinen M. M. Perry M. van Achterberg T. Olde Rikkert M. G. M. Schers H. J. Melis R. J. F. (2015). First experiences with a two-step method for discussing goals with community-dwelling frail older people. Health Expectations, 18(5), 1559-1566. doi:10.1111/hex.12145 26037690 PMC5060876

[bibr35-0898264321993321] Schulman-Green D. J. Naik A. D. Bradley E. H. McCorkle R. Bogardus S. T. (2006). Goal setting as a shared decision making strategy among clinicians and their older patients. Patient Education and Counseling, 63(1-2), 145-151. doi:10.1016/j.pec.2005.09.010 16406471 PMC10791156

[bibr36-0898264321993321] Smit L. C. Schuurmans M. J. Blom J. W. Fabbricotti I. N. Jansen A. P. D. Kempen G. I. J. M. Bleijenberg N. (2018). Unravelling complex primary-care programs to maintain independent living in older people: A systematic overview. Journal of Clinical Epidemiology, 96, 110-119. doi:10.1016/j.jclinepi.2017.12.013 29289764

[bibr37-0898264321993321] Tonigan J. S. Gaume J. Magill M. Hoadley A. Apodaca T. R. Borsari B. Bernstein M. H. (2018). Do what you say and say what you are going to do: A preliminary meta-analysis of client change and sustain talk subtypes in motivational interviewing. Psychotherapy Research, 1-10. doi:10.1080/10503307.2018.1490973 PMC6310665 29954290

[bibr38-0898264321993321] Van Roon E. N. Van Asselt D. Z. B. Vogel D. Van Dijk N. Brouwers R. B. J. (2007). Risk assessment for clinical pharmacists to detect drug related problems in geriatric patients: Results of validation of an easy-to-use triage score system. Basic & Clinical Pharmacology & Toxicology, 101(Suppl. 1), 191-192.

[bibr39-0898264321993321] Vermunt N. P. C. A. Harmsen M. Westert G. P. Olde Rikkert M. G. M. Faber M. J. (2017). Collaborative goal setting with elderly patients with chronic disease or multimorbidity: A systematic review. BMC Geriatrics, 17(1), 1-12. doi:10.1186/s12877-017-0534-0 28760149 PMC5537926

[bibr40-0898264321993321] World Health Organization (2015a). Global Strategy on People-centred and Integrated Health Services - Interim Report. In Service delivery and Safety.

[bibr41-0898264321993321] World Health Organization (2015b). World report on ageing and health.

