# Supplementary material for: Increasing Older Adult Involvement in Geriatric Assessment: A Mixed-Methods Process Evaluation
Source: J Aging Health. 2021 Feb 24;33(7-8):482–92. doi: 10.1177/0898264321993321 (PMC8236665; doi:10.1177/0898264321993321)
Supplement: sj-pdf-1-jah-10.1177_0898264321993321 – Supplemental Material for Increasing Older Adult Involvement in Geriatric Assessment: A Mixed-Methods Process Evaluation [file sj-pdf-1-jah-10.1177_0898264321993321.pdf]

Supplementary Table S1.

Overview of data provided by care professionals and the implementation reach

Care professional	MITI audiotape	Interview	Assessments (n)	Attended Sage-atAge+ MI training sessions
**Nurse A**	-	♦	48	3/3
**Nurse B**	4	♦	113	3/3
**Nurse C**	-	♦	54	0/3*
**Elderly care physician**	1	♦	15	3/3
**Pharmacist**	3	♦	217	0/3*
**Dental care worker**	-	♦	67	1/3
**Allied health care professionals**			57	
**Physiotherapist**	1	♦		3/3*
**Psychologist**	1	♦		0/3*
**Occupational therapist**	-	-		1/3*
**Dietitian**	1	♦		0/3

* Care professional had previously attended training in MI elsewhere

Abbreviations: MI, MI; MITI, MI Treatment Integrity (code)
